# HSP90 Inhibition and Cellular Stress Elicits Phenotypic Plasticity in Hematopoietic Differentiation

**DOI:** 10.1089/cell.2017.0001

**Published:** 2017-10-01

**Authors:** Abdalla A. Lawag, Jennifer M. Napper, Caroline A. Hunter, Nickolas A. Bacon, Seth Deskins, Manaf El-hamdani, Sarah-Leigh Govender, Emine C. Koc, Vincent E. Sollars

**Affiliations:** ^1^ Department of Biomedical Sciences, Joan C. Edwards School of Medicine at Marshall University, Huntington, West Virginia.; ^2^ Department of Natural Sciences, Shawnee State University, Portsmouth, Ohio.

**Keywords:** HSP90, cellular stress, stem cells, cell plasticity, molecular evolution, epigenetics

## Abstract

Cancer cells exist in a state of Darwinian selection using mechanisms that produce changes in gene expression through genetic and epigenetic alteration to facilitate their survival. Cellular plasticity, or the ability to alter cellular phenotype, can assist in survival of premalignant cells as they progress to full malignancy by providing another mechanism of adaptation. The connection between cellular stress and the progression of cancer has been established, although the details of the mechanisms have yet to be fully elucidated. The molecular chaperone HSP90 is often upregulated in cancers as they progress, presumably to allow cancer cells to deal with misfolded proteins and cellular stress associated with transformation. The objective of this work is to test the hypothesis that inhibition of HSP90 results in increased cell plasticity in mammalian systems that can confer a greater adaptability to selective pressures. The approach used is a murine *in vitro* model system of hematopoietic differentiation that utilizes a murine hematopoietic stem cell line, erythroid myeloid lymphoid (EML) clone 1, during their maturation from stem cells to granulocytic progenitors. During the differentiation protocol, 80%–90% of the cells die when placed in medium where the major growth factor is granulocyte–macrophage-colony stimulating factor. Using this selection point model, EML cells exhibit increases in cellular plasticity when they are better able to adapt to this medium and survive. Increases in cellular plasticity were found to occur upon exposure to geldanamycin to inhibit HSP90, when subjected to various forms of cellular stress, or inhibition of histone acetylation. Furthermore, we provide evidence that the cellular plasticity associated with inhibition of HSP90 in this model involves epigenetic mechanisms and is dependent upon high levels of stem cell factor signaling. This work provides evidence for a role of HSP90 and cellular stress in inducing phenotypic plasticity in mammalian systems that has new implications for cellular stress in progression and evolution of cancer.

## Introduction

The process where stem cells become terminally differentiated cells is a complex amalgam of epigenetics, genetics, and the microenvironment. Perturbations in this process are central in a variety of health problems. The theory of field cancerization states that heterogeneity in stem cell populations due to epigenetic or genetic changes can produce perturbations in differentiation. Recent evidence has caused some to postulate that mutations due to replicative stress in stem cells are the primary factor in variation among tissues in the majority of cancers (Tomasetti and Vogelstein, 2015). Understanding how stem cells properly differentiate, despite perturbations, can be elucidated with the application of evolutionary theory.

Canalization is a theory of evolution first postulated by Conrad Waddington that is the process whereby a phenotype is produced despite environmental or genetic variations that may occur (Waddington, 1959). Phenotypes, where canalization is high, have strong robustness or the ability to resist genetic or environmental influences. When canalization is low or absent, phenotypic plasticity occurs allowing a different phenotype to be produced.

If the maturation of stem cells is considered as a canalized event, then factors that influence canalization can affect the ability of stem cells to properly differentiate. If canalization is reinforced, making differentiation more robust, then stem cells may have difficulties selecting from multiple cell fate decisions affected by environmental cues. Reductions in canalization, and thus increases in phenotypic plasticity, could cause transdifferentiation (lineage reprogramming) events or failure in the maturation process. Understanding and manipulation of canalization as it applies to stem cells can have great influences in the fields of regenerative medicine and oncology.

Principal among the factors influencing canalization is Heat Shock Protein 90 (HSP90), a molecular chaperone involved in the cellular stress response. The HSP90 system acts as a protein-folding reservoir that promotes both evolutionary stasis and change. Inhibition of HSP90 has been found to increase the correlation between genotype and phenotype, whereas higher levels of Hsp90 correlate with lower phenotypic variation of inherited mutations (Burga et al., 2011; Casanueva et al., 2012; Jarosz and Lindquist, 2010). HSP90 was first identified as a key molecule in canalization in studies of maize, *Arabidopsis*, and *Drosophila* (Rutherford and Lindquist, 1998). We revealed that HSP90 could operate through an epigenetic mechanism in its role of canalization in *Drosophila* (Sollars et al., 2003). Additionally, HSP90 has been shown to act at the cellular level in the acquisition of traits such as drug resistance in breast tumors (Whitesell et al., 2014).

In this series of experiments, we provide evidence that inhibition of HSP90 produces phenotypic plasticity in an *in vitro* mammalian model of hematopoietic differentiation showing that response to the granulocyte–macrophage colony-stimulating factor (GM-CSF) cytokine is a canalized phenotype. We also provide evidence of a durable change promoting phenotypic plasticity, produced by inhibition of HSP90, and relying upon an epigenetic mechanism most likely involving histone acetylation. Furthermore, we show that various forms of cellular stress can induce similar phenotypic plasticity.

## Methods

### Culture of erythroid–myeloid–lymphoid clone 1 cells

Erythroid–myeloid–lymphoid (EML) cells were obtained directly from Dr. Collins as a gift (Tsai et al., 1994). Low-passage cells are brought from liquid nitrogen storage every 3 months and cultured under standard 5% CO_2_ conditions at 37°C for 2 weeks before use in experiments. Cells are seeded every 2 days at 5.0 × 10^5^ cells/mL in growth medium by centrifugation and resuspension. Growth medium consists of Iscove's modified Dulbecco's medium (IMDM) supplemented with 20% horse serum (ATCC, Manassas, VA), 20% BHK/MKL-conditioned (BHK) medium, penicillin, and streptomycin. Culture growth rates are monitored to evaluate if the culture has shifted from a stem cell culture to that of a progenitor. Standard growth is a cell doubling time of 20 hours. All cultures are fed with fresh growth medium the day before assays.

Assays performed using recombinant stem cell factor (SCF) used either 50 ng/mL (standard) or 25 ng/mL (phase 1 medium) of recombinant murine SCF produced in *Pichia pastoris*, purchased from GenScript (Catalog # Z02997-50). Cells grown for these assays were cultured in growth medium consisting of IMDM supplemented with 10% FBS (Hyclone), 50 ng/mL SCF, penicillin, and streptomycin.

### Selection point assay

EML cells were plated in fresh medium by centrifugation and suspension the day before the protocol. Cultures were treated with the 1.0 nM geldanamycin (EC_50_) at 5.0 × 10^5^ cells/mL for 48 hours. After treatment, cultures were equalized to 5.0 × 10^5^ cells/mL, by centrifugation and resuspension in phase 1 differentiation medium [IMDM with 10 μM all-trans retinoic acid (ATRA; Sigma, St. Louis, MO), 10% BHK-conditioned medium (source of SCF) and 15% WEHI-conditioned medium (source of interleukin-3a), streptomycin, and penicillin] for 2 days. This step must be performed using the yellow light as the only light source to prevent breakdown of the ATRA. Wash the cells with warm PBS three times and then transfer the cells to phase 2 differentiation medium [IMDM with recombinant mouse GM-CSF at 10 ng/mL (BioLegend), streptomycin, and penicillin] and incubate the cells for 24 hours. The following day, stain with Trypan Blue and count the cultures using a hemocytometer.

### Geldanamycin stocks

Geldanamycin was obtained from the NIH and prepared in 40 mM stock solutions in DMSO and stored at −20°C. Stock solutions were discarded after 2 weeks.

### Western blotting

Standard western blotting protocols were performed. The following primary antibodies were used at the manufacturer-specified dilutions: GAPDH (clone 6C5; Fitzgerald), HSP70 (clone C92F3A-5; Enzo Life Sciences), and ERK 1/2 (clone 137F5; Cell Signaling Technology). Secondary antibodies, including polyclonal goat anti-rabbit (Bio-Rad) and polyclonal sheep anti-mouse HRP-conjugated secondary antibodies (GE Healthcare Life Sciences) were used along with SuperSignal™ West Pico Chemiluminescent Substrate (Thermo Fisher Scientific) for visualization. Eighty micrograms were loaded in each lane.

### Functional assay for SCF

Each lot of BHK medium was tested for functionality by an overnight assay using standard growth medium augmented with either the normal level of 20% BHK medium or 10%. Wells were seeded at 5.0 × 10^5^ cells/mL and assayed for culture growth with Trypan Blue live/dead assays. Lots where 20% BHK medium did not support robust culture growth (at least culture doubling) were discarded.

### Flow cytometry analysis

EML cells were prepared by washing once with PBS followed by incubation with live/dead stain, Zombie-NIR (BioLegend, cat# 77184), according to the manufacturer's protocol. Cells were then washed once in FACS buffer (PBS supplemented with 0.5% bovine serum albumin and 2 mM EDTA) and collected by centrifugation at 300 *g*. Cells were washed again and labeled with antibodies for 30 minutes on ice in FACS buffer. Cells were washed after antibody incubation once and immediately processed for analysis on the flow cytometer. The following antibodies from BioLegend were used in the initial characterization of EML cells: PE-conjugated Sca-1 (clone D7), APC-conjugated CD11b (clone M1/70), PE-conjugated F4/80 (clone BM8), APC-conjugated CD117 (clone 2B8), PE-conjugated Ly-6G (clone RB6-8C5), and PE-Cy7-conjugated Ter119 (clone Ter119).

The following antibodies from BD Biosciences were used in the MPP studies: BB515-conjugated Sca-1 (clone D7), PE-CF594-conjugated CD117 (clone 2B8), APC-conjugated CD48 (clone HM48-1), PE-conjugated CD135 (clone A2F10.1), and BV421**-**conjugated CD150 (clone Q38-480). Compensation controls were created using eBioscience UltraComp eBeads (cat# 01-2222-92) for antibodies or EML cells with Zombie-NIR for the live/dead stain. Data acquisition was performed using BD FACSAria I flow cytometer/sorter. Data analysis/compensation was performed using FlowJo v. 10.1 software (TreeStar, Ashland, OR) with fluorophore-matched isotype controls to ascertain nonspecific binding, and fluorescence minus one controls for setting gates during analysis. Superenhanced Dmax subtraction analysis was used for determination of differences in histograms that were not sufficiently separated.

### Cellular stress protocols

For hypoxic stress, cultures were incubated in 1% oxygen and 5% CO_2_ for 8 hours (New Brunswick Galaxy 170 incubator) then transferred back to ambient incubation for 16 hours repeatedly for two cycles (Cooper et al., 2004). Cells were allowed to recover for 48 hours. For hydrogen peroxide stress, cultures were treated with 100 μm hydrogen peroxide for 24 hours, provided fresh medium, and given another 24 hours for recovery (Wang et al., 1998). For oxidative stress, cultures were treated with 40 mm glucose for 24 hours, followed by washing with warm PBS, and given 24 hours for recovery (Ding et al., 2007). For serum starvation stress, cultures were seeded with growth media lacking serum for 24 hours, then provided fresh medium, and rested for 24 hours. In all the cellular stress protocols, cultures were placed on the selection protocol after the rest period.

### Protein carbonylation assay

Whole cell lysates were prepared with lysis buffer containing 50 mM Tris-HCl (pH7.6), 150 mM NaCl, 1 mM EDTA, 1 mM EGTA, 1% Nonidet P-40, 0.1% SDS, 1 mM PMSF, and proteinase and phosphatase inhibitor cocktails. Equal amounts of total protein (20 μg) were denatured at 6% SDS and derivatized with 1 × DNPH (100 mM in 10 N HCl, 2,4-dinitrophenylhydrazine, Sigma-Aldrich) to form DNP hydrazine derivatives and neutralized with buffer containing 30% glycerol in 2 M Tris base, as previously described (Yan et al., 2013). Derivatized protein samples (20 μg) were run on 10% SDS-PAGE and probed with anti-DNP antibody (Sigma-Aldrich) at 1:20,000 dilution. Protein carbonylation detected below 55 kDa was used for densitometry analysis.

### Statistical analyses

Statistical analyses were performed using GraphPad Prism 7. Student's *t*-type tests were performed with simple comparisons. Multiple comparisons were performed with ANOVA analysis, tests for normal distribution, and Student's *t*-type tests with the Dunnett's correction for multiple comparisons when comparing to controls, Tukey's correction when making all possible comparisons, or Sidak's correction when using paired samples (geldanamycin and sodium butyrate treatments). Reported *p*-values represent these values after correction for multiple comparisons.

## Results

### Inhibition of HSP90 causes a durable change in EML cells resulting in a selective advantage

**FIG. 1.**
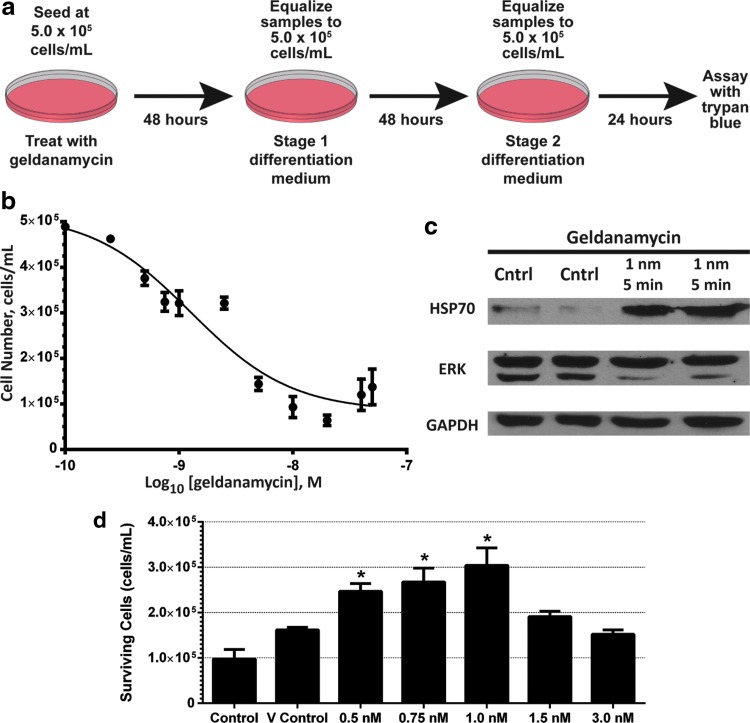
Inhibition of Hsp90 in EML cells. **(a)** A schematic showing the protocol of the selection point assay using EML cells. **(b)** EML cells were treated with geldanamycin for 24 hours followed by Trypan Blue staining. A logarithmic plot of the dose–response curve showing that the EC_50_ of geldanamycin in EML cells is 1.1 nM. **(c)** Representative western blots showing activation and HSP70 and inhibition of the HSP90 client protein ERK 2 (upper band is ERK 1, lower is ERK 2). Control cells are treated with the DMSO vehicle. **(d)** The mean with SEMs of cells surviving the selection point as determined by Trypan Blue staining, after the 5-day protocol, is displayed with the untreated control, the DMSO VC, and the various treatment levels of geldanamycin. Data represent six independent experiments. *Denotes significant differences compared with the VC with a *p*-value less than 0.0001. EML, erythroid myeloid lymphoid; VC, vehicle control.

To test for loss of canalization and induction of phenotypic plasticity, we are using an *in vitro* system incorporating selective pressure and measurable adaptability. We have obtained the EML cell line from its creator Dr. Tsai. EML cells are a suspended murine hematopoietic stem cell (HSC) line comprising mostly blast-appearing cells that can be induced to differentiate into myeloid or lymphoid cells (Tsai et al., 1994). This cell line is SCF dependent and has been immortalized by overexpression of a dominant-negative retinoic acid receptor. By inducing these cells to differentiate into macrophages and granulocytes by the sequential addition of IL-3 (interleukin 3, stage 1 differentiation medium) and GM-CSF (stage 2 differentiation medium), we have found that 70%–90% cellular death occurs when the culture is switched to medium containing GM-CSF as the primary survival factor (Fig. 1A). We term this point, where much of the culture undergoes death, as the selection point.

By using this selection point assay, we can measure changes in the ability of EML cells to survive the selective pressure of survival in GM-CSF medium. Increased survival indicates that the cells normally undergoing death are able to change their phenotype conducive to survival with stimulation by GM-CSF and are thus exhibiting phenotypic plasticity.

In our selection point assay, we used geldanamycin as an inhibitor of HSP90. Use of this pharmacological inhibition rather than genetic or RNA interference–mediated depletion of HSP90 protein allowed us to expose cells to mild modulation of the chaperone activity over a short term, thereby avoiding effects on cell viability and compensatory expression of the two HSP90 isoforms. Geldanamycin is used because of its short half-life in aqueous solutions to produce the most transitory inhibition (Carreras et al., 2003; Supko et al., 1995).

Using culture growth assays over a 24-hour period, we identified the EC_50_ level of geldanamycin in EML cells as measured by culture death as 1.1 nm (Fig. 1B). Levels as low as 5 nM have extreme effects on cell survivability. The EC_50_ level of geldanamycin causes induction of HSP70 and loss of the HSP90 client protein ERK 2, verifying that inhibition of HSP90 is occurring (Fig. 1C). The standard method for showing downregulation of HSP90 protein activity is to show downregulation of a client protein and upregulation of HSP70 (Do et al., 2015; Powers et al., 2013). This geldanamycin treatment level corresponds to levels of the most sensitive cell types, such as HER-2-positive breast cancer cells. Typical primary murine cells have EC_50_ levels of 600 nm or higher. Lower EC_50_ levels of inhibition of HSP90 by geldanamycin-based inhibitors have been attributed to a lower level of unbound HSP90, which is typically present in cancer cells (Kamal et al., 2003).

Forty-eight hours before inducing differentiation, EML cells were treated with geldanamycin and allowed to recover. Transient inhibition of HSP90 by geldanamycin causes a dose–responsive increase in cell survival 5 days later through the selection point (Fig. 1D). Levels of geldanamycin as low as 0.5 nM caused significant increases in cell survival compared with the vehicle control (VC) with a 150% increase compared with the untreated control. This survival increased to as much as three times the untreated control value as the dose increased, but was lost once levels rose over the EC_50_ level. At 1.5 and 3.0 nM, the number of cells surviving the selection point was similar to the VC. Since the cell doubling time of EML cells is approximately 20 hours and the selection point occurs 96 hours after treatment with geldanamycin, the transient inhibition of HSP90 caused a durable change that was persistent through several cell division cycles. Thus, inhibition of HSP90 resulted in increased plasticity in this mammalian model system, suggesting survival through the selection point of GM-CSF medium is a canalized trait.

### Characterization of myeloid cell differentiation during the selection point assay

**FIG. 2.**
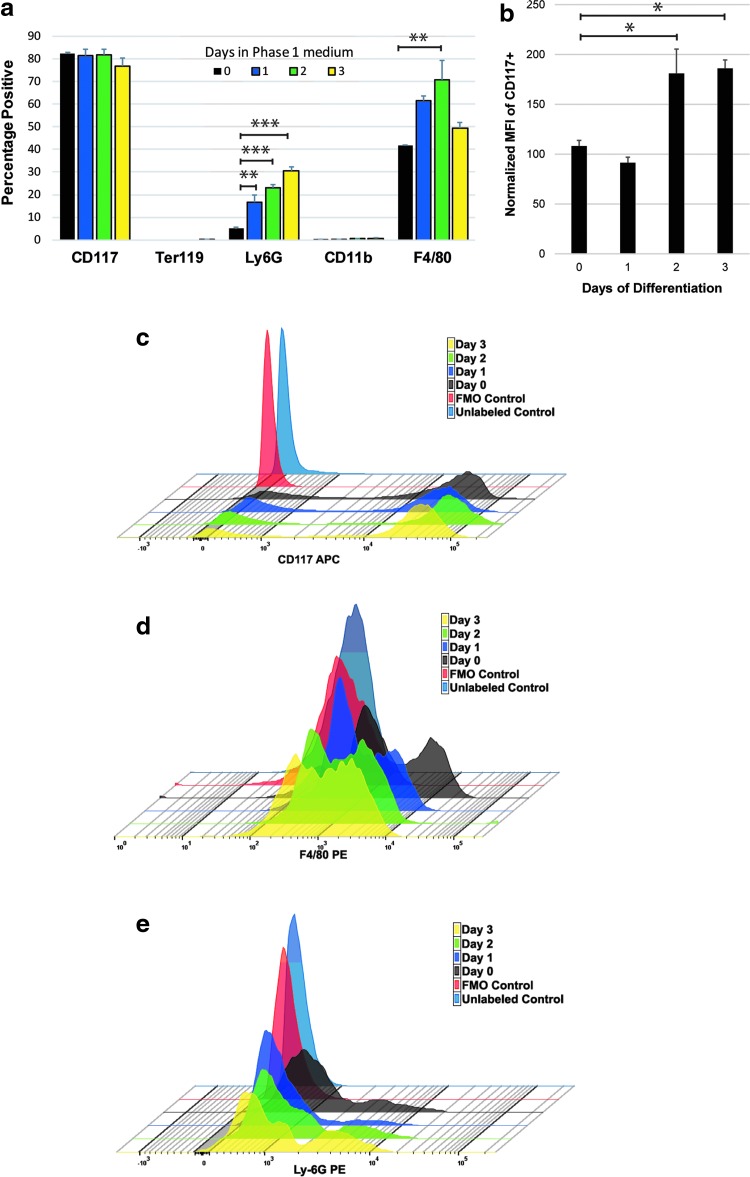
Characterization of the selection point. Analysis of differentiation with immunophenotyping using flow cytometry, over the course of 3 days, after placing EML cells in phase 1 differentiation medium containing IL-3, SCF, and all-trans retinoic acid. **(a)** EML cells analyzed for the percentage of cells expressing various markers of differentiation. **(b)** The MFI of the CD117 marker for progenitor and stem cells. The MFIs of CD117 increase as EML cells differentiate and reach a peak during the time of selection. **(c–e)** Representative histograms for each data set where significant levels of each marker are seen. *p*-values that are less than 5% after corrections for multiple comparisons are represented by (*, ** are *p*-values <0.01, *** are *p*-values <0.001). Analysis represents an N of 4 or more with error bars delineating SEM values. MFI, median fluorescent intensity.

To characterize the differentiation process occurring during our selection point assay, immunophenotyping was performed using various markers of hematopoietic differentiation (Fig. 2). Cells were placed in stage 1 differentiation medium and monitored daily with flow cytometry. Flow cytometry data for CD117 (c-kit), Ter119 (a marker for erythroid differentiation), Ly6G (a general marker for granulocyte–monocyte differentiation), CD11b (a marker for terminal granulocyte differentiation), and F4/80 (a marker for macrophage differentiation) were analyzed (Fig. 2A). Although the percentage of CD117^+^ cells remained constant at about 80%, we did find that the MFI (median fluorescent intensity) increased significantly starting at day 2 (Fig. 2B, C). This increase in CD117 expression levels seen as EML cells reach the selection point agrees with data obtained *in vivo* correlating proliferation rates of HSCs with expression rates of CD117 (Sawen et al., 2016; Shin et al., 2014).

These studies found that higher CD117 expression levels were found in the more mature HSC populations and those at a higher proliferation rate. The rise in CD117 expression we see as the differentiation protocol proceeds is consistent with EML cells differentiating into a more mature cell type. F4/80^+^ cells also had a transient increase of about 75% that peaked at day 2 (the selection point, Fig. 2D). Ly6G^+^ cells (from 5% to 30%) were found to significantly increase as differentiation proceeded over the course of 3 days (Fig. 2E). Both Ter119 (a marker of erythroid maturation) and CD11b (a later-stage granulocyte marker) markers were not found in the differentiating cell population. Sca-1 levels were also tested, but remained constant at ∼70% throughout differentiation (data not shown).

These data indicate that the culture conditions being used are inducing differentiation into granulocyte and macrophage lineages, with the culture maintaining early stem/progenitor characteristics.

### Inhibition of HSP90 does not change the immunophenotype of differentiating EML cells

**FIG. 3.**
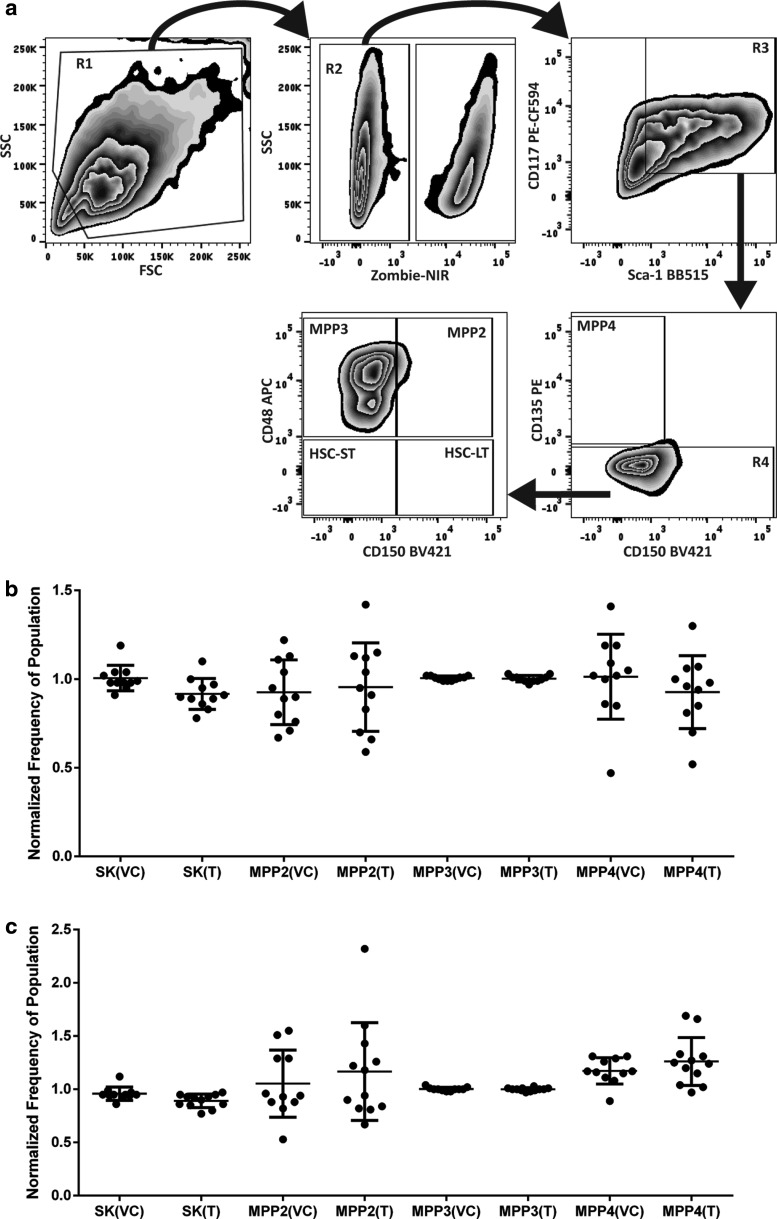
Immunophenotype of cells remain unchanged at the selection point. **(a)** Gating strategy used in the analysis of MPP subtypes. EML cells were first sorted based upon forward scatter and side scatter properties (R1) and then on a live/dead stain (R2) to identify live single cell populations. Cells were then gated to select cells positive for both the CD117 and Sca-1 antigens (R3). The CD135 and CD150 antigens were used to select cells conforming to the MPP4 subtype and R4. Cells matching the R4 gating parameters were gated based upon CD48 and CD150 to determine MPP2, MPP3, and hematopoietic stem cell subtypes. **(b)** The normalized frequencies of the Sca-1+ and C-kit+ (SK) populations, the MPP2, MPP3, and MPP4 cell populations at Day 0 of the selection point experiments and **(c)** Day 2. The distribution of the data (*n* = 11) are illustrated with SDs for the VC and treated samples (T) normalized to the untreated controls (not displayed). Comparisons between vehicle and treated samples using the Student's *t*-test showed no significant differences. MPP, multipotential progenitor.

One possible mechanism that could explain increased survival through the selection point is that inhibition of HSP90 may alter the immunophenotype of EML cells such that a larger portion of them have a phenotype amenable to GM-CSF stimulation. EML cells in the selection point assay were subjected to immunophenotyping to determine if inhibition of HSP90 changed the subtypes of stem and progenitor cells in the population (Fig. 3). Cells were immunophenotyped with established antigens in murine systems, including Sca-1, CD48 (SLAMF2), CD117, CD135 (FLK2), and CD150 (SLAMF1) to determine their subtypes of multipotential progenitors, MPPs [(Fig. 3A), (Akala et al., 2008; Oguro et al., 2013; Pietras et al., 2015; Wilson et al., 2008)].

The EML cell cultures were exclusively of the MPP3 subtype (Sca-1^+^, CD117^+^, CD48^+^, CD150^−^, CD135^−^), which did not change substantively with the inhibition of HSP90 (Fig. 3B) or during the course of the 2 days of differentiation in our assay (Fig. 3C). Comparisons of VC to geldanamycin-treated frequencies of Sca-1^+^CD117^+^, MPP2 (Sca-1^+^, CD117^+^, CD48^+^, CD150^+^, CD135^−^), MPP3, MPP4 (Sca-1^+^, CD117^+^, CD48^−^, CD150^−^, CD135^+^), or HSC subtypes found no significant differences in frequencies of these stem and progenitor subtypes. This subtype of MPPs is primed for differentiation into myeloid progenitor cells (Pietras et al., 2015). These data indicate that no shift in immunophenotype as assayed by these markers of the cell population with inhibition of HSP90 occurs, and thus this does not explain the increased ability of cells subjected to inhibition to survive the selection point. This does not rule out subtle changes in differentiation potential of these cells.

### Inhibition of HSP90 does not change the proliferation rate of differentiating EML cells

**FIG. 4.**
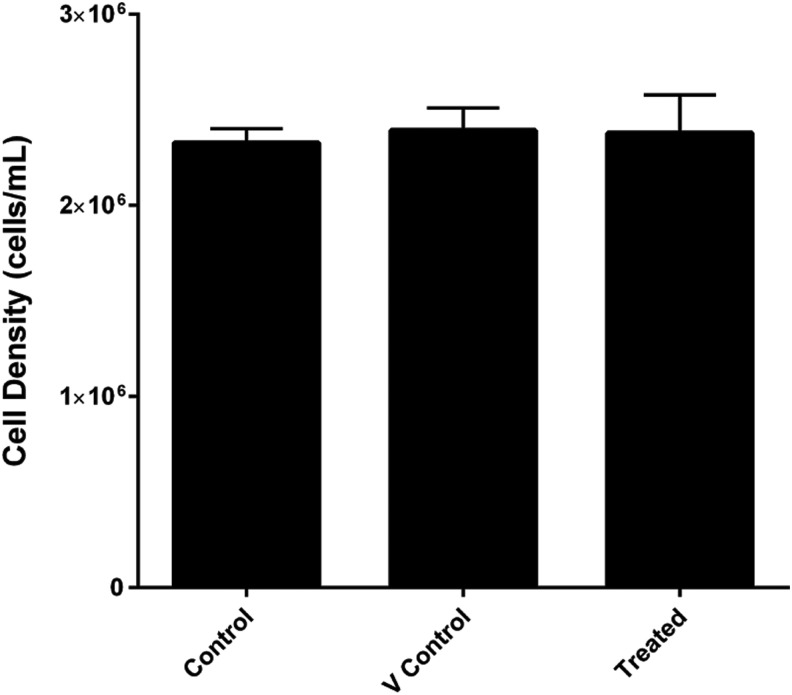
HSP90 inhibition does not alter the proliferation rate of differentiating EML cells. A comparison of growth rates of cultures of untreated controls, VCs, and geldanamycin treated (1.0 nM) after 2 days in stage 1 differentiation medium using Trypan Blue live/dead stain. Error bars represent SEMs. Graphed data represents an *N* = 8.

To determine if increased survival through the selection point could be explained by an increase in proliferation rate caused by transient inhibition of HSP90, culture growth assays were performed. Growth of differentiating cultures was assayed by Trypan Blue assays (Fig. 4). These cultures followed the standard EML selection point protocol, starting at 5.0 × 10^5^ cells/mL. All of them experienced robust culture growth after 2 days with over a four-fold increase during the assay, with the untreated control ending with 2.3 ± 0.2 × 10^6^ cells/mL. No significant differences were found in any of the experimental groups indicating inhibition of HSP90 does not significantly alter culture growth rates.

### Increased phenotypic plasticity is dependent upon high levels of SCF

**FIG. 5.**
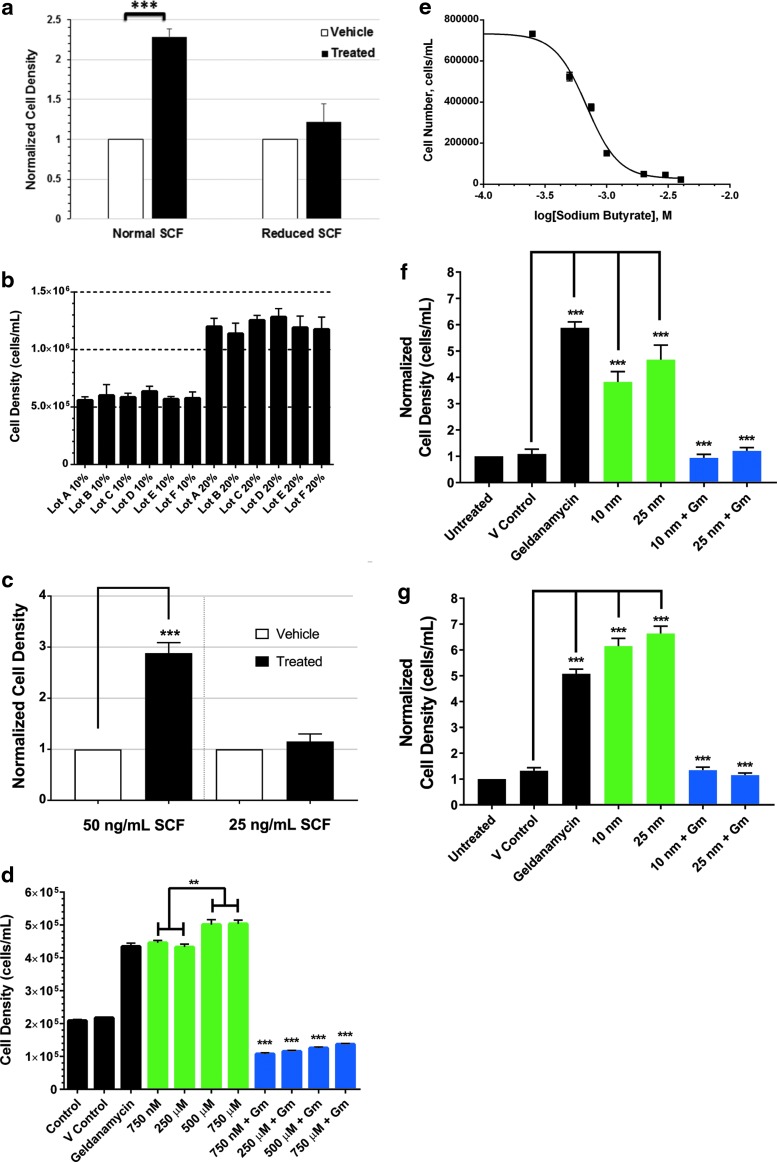
The selection point effect in EML cells is dependent upon high-level signaling through SCF receptor and histone acetylation. **(a)** Selection point experimental data, where cells are exposed to EC_50_ levels of geldanamycin at −48 hours with either normal (20%) or reduced levels (12.5%) of SCF provided in conditioned medium. The remaining aspects of the protocol remain unchanged. Cell counts were normalized to the VC. These data represent at *N* = 13 for normal SCF and 11 for reduced SCF. **(b)** A functional assay conducted on six lots (lots A–F) of BHK-conditioned medium containing SCF using 10% BHK-conditioned medium or 20% to stimulate EML cells. Cells were seeded at 5.0 × 10^5^ cells/mL and stimulated with growth medium containing BHK-conditioned medium for 24 hours. Trypan Blue was used to assay culture growth. **(c)** Selection point experiments using recombinant SCF during culturing of the EML cells and the entire protocol instead of BHK-conditioned medium. The levels of SCF were varied only during the geldanamycin incubation step. **(d)** Selection point experimental data using various levels of sodium butyrate (*green bars*) or geldanamycin with sodium butyrate (*blue bars*). *p*-value designations above the *green bars* represent those between various levels of sodium butyrate, whereas those above *blue bars* represent comparisons with the corresponding sodium butyrate only control. Data presented represent an *N* = 9. **(e)** EML cells were treated with sodium butyrate for 24 hours followed by Trypan Blue staining. A logarithmic plot of the dose–response curve showing that the EC_50_ of geldanamycin in EML cells is 689 μM. Experiments similar to **(d)** with **(f)** vorinostat or **(g)** belinostat. Error bars represent SEMs. Significance levels of Student's *t*-type tests for all panels represent *p* < 0.01 (**) or *p* < 0.001 (***). SCF, stem cell factor.

SCF binds and activates the receptor tyrosine kinase class III c-Kit, and this interaction is critical for normal hematopoiesis and survival of EML cells. In our *Drosophila* studies, we found that ectopic expression of Wingless occurred because of inhibition of HSP83, the *Drosophila* homolog of mammalian HSP90 (Sollars et al., 2003). To test if HSP90 is interacting with the signal transduction cascades in the EML selection point model, we varied the levels of SCF in the medium during the geldanamycin exposure step (Fig. 5A). During this first 48 hours of the experiment, SCF levels were used at normal levels (20% conditioned medium) or at reduced levels (12.5%). Afterward, SCF levels were returned to normal levels during the differentiation portion of the protocol. We found that reduction of SCF levels to 12.5% during geldanamycin treatment caused a loss of selective advantage once the cells reach the selection point.

Our data in this assay show that with normal SCF levels during geldanamycin treatment there is a two-fold increase in cells at the selection point, while if we reduce SCF levels to 12.5% the number of cells surviving selection returns to vehicle-treated levels (Fig. 5A). This suggests that high-level signaling through the SCF receptor is necessary for the phenotypic plasticity induced by inhibition of HSP90.

Functional assays were performed on various lots of BHK-conditioned medium to ascertain their effects on EML cells at high and low concentrations (Fig. 5B). Cells were seeded at 5.0 × 10^5^ cell/mL in either medium containing 10% or 20% BHK-conditioned medium, and cultures were assayed for growth with Trypan Blue live/dead stain. Cultures seeded with 10% BHK-conditioned medium maintained their density, but did not proliferate normally. Cultures seeded with 20% BHK-conditioned medium grew normally with a greater than two-fold increase in cells. Variance between lots was nominal in this functional assay. The differential between these two supplement levels indicates that low levels of SCF in the medium are sufficient for EML cell survival, whereas high levels are necessary for proliferation.

To rule out constituents other than SCF present in BHK-conditioned medium being responsible for the effects on the selection point, we used selection point assays with recombinant SCF (Fig. 5C). These assays were performed with cells that were grown in recombinant SCF and FBS for at least 2 weeks, before performing the selection point assay. Results were similar to those obtained with BHK-conditioned medium, where 50 ng/mL was sufficient to propagate the culture and induce a selection point advantage, whereas 25 ng/mL did neither of these.

### HSP90-mediated phenotypic plasticity is inhibited by sodium butyrate

The increased plasticity induced by inhibition of HSP90 is durable through several mitoses (the doubling rate of EML cells is 20 hours). Since we have found histone acetylation to be a necessary mechanism for HSP90-elicited epigenetic effects, sodium butyrate treatment was used to inhibit histone deacetylases (HDACs) in the EML selection model (Sollars et al., 2003). The selection point was conducted as in Figure 1A, except 24 hours after treatment with geldanamycin or vehicle, sodium butyrate was added to the culture (Fig. 5D). Sodium butyrate alone caused increased survival at the selection point at all levels tested (green bars) to levels similar to that occurring due to inhibition of HSP90. When treatment levels of sodium butyrate rise to 500 μM and above (close to the EC_50_ levels in EML cells, Fig. 5E), there is a modest (10%) but significant increase in cells surviving the selection point.

Statistical analysis of cell survival data indicates that there are significant differences between the 750 nM or 250 μM and the 500 μM or 750 μM treatment levels (Fig. 5D). This suggests that alterations in the ability of EML cells to effectively use histone modification in epigenetic gene expression can induce phenotypic plasticity. The further increase as EML cells is treated with sodium butyrate levels that result in cellular stress (those reaching the EC_50_ of sodium butyrate) suggests that cellular stress can enhance this effect.

When we combined the treatments of sodium butyrate with geldanamycin, we found that inhibition of HDACs eliminated the increased survivability of cells at the selection point induced by inhibition of HSP90 (Fig. 5D). Comparisons of sodium butyrate-treated culture selection point survival rates to those treated with 1.0 nM geldanamycin as well, found significant differences at all levels of sodium butyrate treatment. This indicates that increased plasticity of the cell population can be induced by an epigenetic mechanism and that increased plasticity induced by inhibition of HSP90 requires histone acetylation. We also performed these experiments using the more selective HDAC inhibitors vorinostat (Fig. 5F) and belinostat (Fig. 5G) with similar results as sodium butyrate. It is also worthwhile to note that HDAC inhibitors can induce hyperacetylation of Hsp90 and block binding to substrates (Nimmanapalli et al., 2003; Yu et al., 2002). However, our treatments are separated by 24 hours in this assay.

### Cellular stress induces phenotypic plasticity

One of the primary roles of HSP90 is to protect cells from environmental and chemical stresses. Cellular stress will put demands on HSP90 present in the cell, typically 1%–2% of total cellular protein, but up to 5% in transformed cells (Lai et al., 1984; Wang et al., 2010). This can limit the ability of HSP90 to perform its normal functions, potentially acting as an inhibitor of HSP90 function. Additionally, cellular stresses have been found to adversely affect HSP90 protein levels in conditions of H_2_O_2_ exposure, oxidative stress, free radical exposure, and UV exposure (Beck et al., 2012; Chen et al., 2009; Panopoulos et al., 2005; Pantano et al., 2003).

**FIG. 6.**
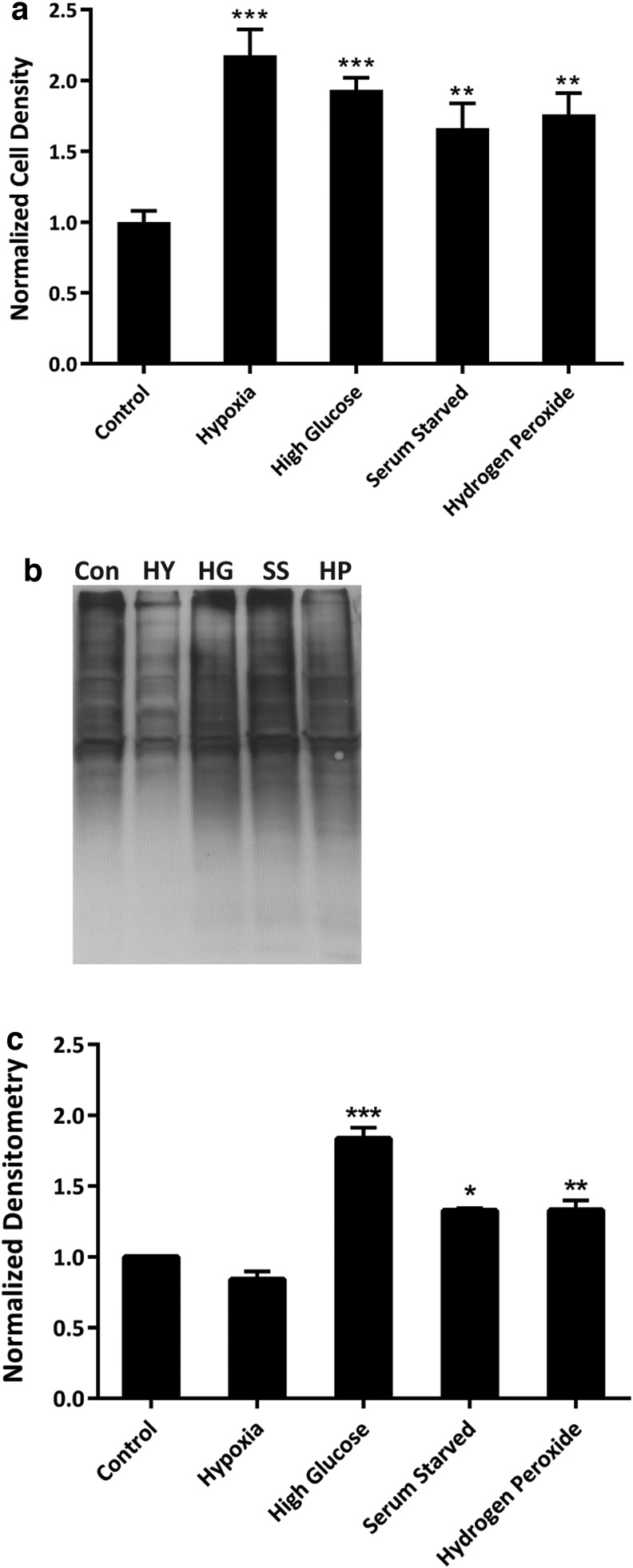
Cellular stress protocols produce similar results to Hsp90 inhibition. **(a)** The cell densities of cultures subjected to four cellular stress protocols and then subjected to the selection point assay. Cell counts were normalized to control cells that were untreated. **(b)** A representative blot of protein carbonylation assays converted to DNP hydrazine derivatives that were performed on EML cells subjected to the various stress protocols. **(c)** Densitometry analysis of protein carbonylation assays. Error bars represent SEMs with an *N* = 9 for the selection point experiments and *N* = 3 for the protein carbonylation assay. Comparisons to the control value are shown where the *p*-value was less than 0.05 (*), 0.01 (**), or 0.0001 (***). HY, hypoxia; HG, high glucose; SS, serum starvation; HP, hydrogen peroxide.

To determine if cellular stress could induce phenotypic plasticity we performed a variety of cellular stress protocols utilizing H_2_O_2_ exposure, oxidative stress, hypoxia, and serum depravation followed by a recovery period before utilizing our EML selection point assay (Fig. 6). The results indicate that all cellular stress protocols produced significant levels of increased survival in the assay (Fig. 6A). These levels were comparable to that produced by direct inhibition of HSP90 by geldanamycin (Fig. 1D), with 60%–110% increases in cell survival depending upon the cellular stress protocol used.

To verify that the protocols performed elicited cellular stress, protein carbonylation assays were performed to measure production of protein oxidation (Fig. 6B, C). The hypoxia levels resulted in low levels of oxidized proteins, presumably due to low oxygen levels of the stress, and the highest induction of phenotypic plasticity according to the selection point assay (Fig. 6A, C). Oxidized protein levels were significantly higher after the high glucose, serum starvation, and hydrogen peroxide cellular stress protocols (Fig. 6C). The levels of oxidized proteins for these samples also correspond to the levels of plasticity induced in the selection point assay (Fig. 6A, C), with the highest levels of surviving cells found in the high glucose group and the other two being roughly equal.

## Discussion

**FIG. 7.**
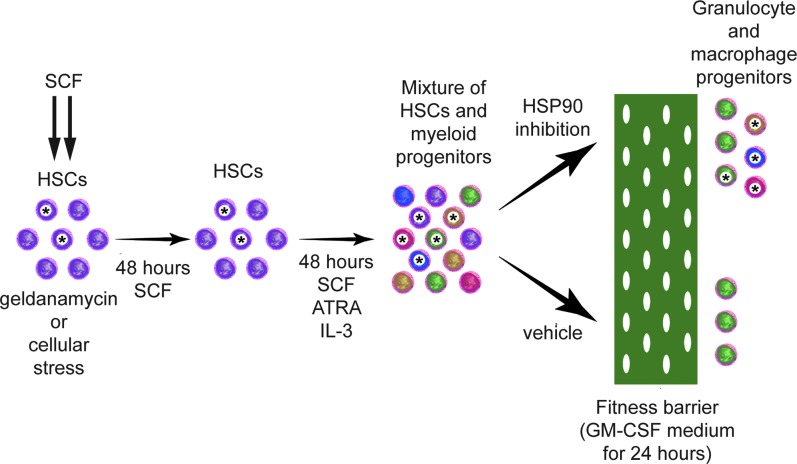
EML cell selection point model. EML cells model the capabilities of HSCs. In the selection model, we treat the cells with geldanamycin to inhibit HSP90, but our results also indicate that cellular stress can affect the selection point similarly. This induces a durable change in a portion of the cells (*asterisks*) that is epigenetic in nature. After a recovery period, the first stage of differentiation is begun, including all-trans retinoic acid to remove the blockage to differentiation produced by the dominant negative retinoic acid receptor transgene in this cell line, as well as SCF and Interlekin-3 (IL-3) to induce myeloid differentiation. After 2 days, a mixture of cell types is produced that are transferred into the second-stage differentiation medium containing GM-CSF as the major cell survival agent. This situation models a selection event where the fitness barrier is the capacity to survive in the new medium (*green* field with holes). Normally, only cells who “match” the fitness barrier (*green* cells with *green* fitness barrier) pass through and survive as in the case of the VC treatments. Our data show that inhibition of HSP90 increases cell survival by as much as three fold. We hypothesize that the HSP90 inhibition-induced epigenetic change increases cellular plasticity by reducing canalization resulting in increased phenotypic variability of the cells. The plasticity allows the cells that have similar cell fates (*blue*, *red*, and *yellow* cells) as the ones normally able to pass through the fitness barrier, to more closely match the barrier, or to adapt to more closely match the barrier. GM-CSF, granulocyte–macrophage colony-stimulating factor; HSC, hematopoietic stem cell.

These experiments have demonstrated that the EML selection point model displays a phenotype of a plastic nature that is responsive to inhibition of HSP90 (Fig. 7). While the possibility exists that mitosis is a key factor in the durable changes inhibition of HSP90 elicits, there is also the possibility that other effects of differential signaling through the SCF receptor are the key factor. Differential signaling through the SCF receptor has long been established to be manifested in activation of the various signal transduction cascades under this receptor (PI3-kinase, Src family kinases, mitogen-activated protein kinase pathways, and phospholipases), its interaction with other cytokine receptors (IL-3, G-CSF, GM-CSF, and EPO), and turnover of the receptor (reviewed in Lennartsson and Ronnstrand, 2012).

What mechanism is critical and interacting with HSP90 inhibition remains to be seen, but several signaling intermediates in these pathways are known to be HSP90 clients, including but not limited to ERK and AKT (Fontana et al., 2002; Setalo et al., 2002). We are currently investigating the effects of HSP90 inhibition on signal transduction intermediates in this model.

Cellular stress can induce changes similar to those induced by geldanamycin treatment to cell survivability at the selection point. It remains to be determined the mechanism by which cellular stress induces phenotypic plasticity, and to replicate these results in other models of phenotypic plasticity. Although, in our experiments there was a correlation between oxidative damage and survival through the selection point, the highest level of survival was seen with hypoxia stress. HSP90 is known to interact with the major orchestrator of acute hypoxia, hypoxia-inducible factor-1 alpha (Isaacs et al., 2002).

These results along with its demonstrated role in promoting survival through the selection point suggest that the role of HSP90 in cellular stress is the mechanism whereby cellular stress induces phenotypic plasticity. Perhaps it is through allocation of HSP90 to recovery from the stress conditions, and a corresponding loss of HSP90 function to roles related to phenotypic plasticity. Determining the mechanism of the interaction between phenotypic plasticity, cellular stress, and HSP90 will give important insights into molecular evolution of tumors.

Three lines of argument support the role of epigenetic gene regulation in the form of histone acetylation as the mechanism for the exhibited phenotypic plasticity. First, our previous experiments in the *Drosophila* model system showed a connection between *Hsp83* (the *Drosophila* ortholog of *Hsp90*), *trithorax* group genes, transgenerational epigenetic inheritance, and histone acetylation (Sollars et al., 2003). The similar roles of HSP90 as a canalizer of phenotypic plasticity in the *Drosophila* model and those presented in this study suggest the same mechanism of action.

Second, the time delay between administration of geldanamycin and the elicitation of the phenotypic plasticity (5 days and five cellular divisions) suggests a durable mechanism of cellular memory. The epigenetic events that are initiated in this model are dependent upon a high level of signaling through the SCF receptor at the time of initiation, but are durable through several mitosis events.

Third, the experiments with HDAC inhibitors detailed in this study show the ability to induce phenotypic plasticity alone, but in combination with geldanamycin result in a loss of plasticity. Investigating the amount of conservation of HSP90-mediated canalization of cellular phenotypes in mammalian systems from other model systems will be instrumental in our understanding of molecular evolution.

Understanding and exploiting the process of canalization at the cellular level can lead to new therapies on diverse human health concerns through a variety of mechanisms. For instance, the molecular evolution of cancers both before and after treatment is widely recognized. Examining the effects of low-level inhibition of HSP90 has been found to affect the acquisition of resistance to hormonal therapies in breast cancer models, perhaps through affecting phenotypic plasticity (Whitesell et al., 2014). Understanding the interactions between canalization and the acquisition of phenotypes of cancers will have important ramifications for HSP90 inhibitors in clinical trials. Furthermore, our understanding of the interaction of cellular stress and phenotypic plasticity in human homeostasis can lead to a deeper understanding of cellular differentiation.

Analysis of transcription networks in human macrophages has demonstrated a much more diverse set of cell types than the M1 (inflammatory) versus M2 (repair) classes, indicating that macrophages have a high level of phenotypic plasticity (Xue et al., 2014). Examining the role of canalization in the differentiation of macrophages could have important ramifications in diseases, such as COPD, obesity, cancer, and asthma. Moreover, dedifferentiation of progenitor cells into stem cells has been found to occur in multiple cell types and may be a canalized phenotype that can be increased with inhibition of HSP90 (Rompolas et al., 2012; Tata et al., 2013; Tetteh et al., 2015; van Es et al., 2012; Yanger et al., 2014).

If true, this could have ramifications to our understanding of cancer progression in both the acquisition of cell immortality similar to stem cells and resistance to chemotherapies that more dedifferentiated tumors typically have as a characteristic. Additionally, the ability to produce stem cells from more differentiated cells and better control their selection of cell fates would increase the pool of cells available for regenerative medicine and tissue engineering.

As Drs. Feinberg and Pujadas postulated, it is expected that the degree of variability (or noise in their terms) at the epigenetic level is expected to be higher both with increased pluripotency and during the process of cell fate determination (Pujadas and Feinberg, 2012). Furthermore, the picture emerging from studies of noisy systems is that they offer the advantage of high phenotypic plasticity (Eldar and Elowitz, 2010; Raj and van Oudenaarden, 2008).

The studies detailed here provided evidence that one of the sources of plasticity in these systems is in response to cell signaling and that cellular stress can increase plasticity during cellular differentiation. The results of these studies have the limitation of being derived from a single model in one noncancerous cell line, and thus potentially prone to artifacts of the cell line. Thus, replicating these results in other models of plasticity needs to be performed. Investigating the role of HSP90 in controlling cellular plasticity, in terms of the ability of cells to make changes to their cellular fates, using the EML cell model will provide a rich model effectively modeling canalization in a mammalian stem and progenitor cell system.

## Author Contributions

Abdalla A. Lawag: Collection and/or assembly of data, data analysis and interpretation, and article writing.

Jennifer M. Napper: Conception and design, collection, and/or assembly of data.

Caroline A. Hunter: Collection and/or assembly of data, and data analysis and interpretation.

Nickolas A. Bacon: Collection and/or assembly of data, and data analysis and interpretation.

Seth Deskins: Collection and/or assembly of data, and data analysis and interpretation.

Manaf El-hamdani: Collection and/or assembly of data.

Sarah Govender: Collection and/or assembly of data.

Emine Koc: Conception and design, collection and/or assembly of data, and data analysis and interpretation.

Vincent E. Sollars: Conception and design, collection and/or assembly of data, data analysis and interpretation, article writing, and final approval of the article.
